# Full-Length Transcriptome Sequencing Reveals Alternative Splicing and lncRNA Regulation during Nodule Development in *Glycine max*

**DOI:** 10.3390/ijms23137371

**Published:** 2022-07-01

**Authors:** Jing Liu, Shengcai Chen, Min Liu, Yimian Chen, Wei Fan, Seunghee Lee, Han Xiao, Dave Kudrna, Zixin Li, Xu Chen, Yaqi Peng, Kewei Tian, Bao Zhang, Rod A. Wing, Jianwei Zhang, Xuelu Wang

**Affiliations:** 1 College of Life Science and Technology, Huazhong Agricultural University, Wuhan 430070, China; gingel77@163.com (J.L.); chenshengcai2012@163.com (S.C.); lmlmlm@webmail.hzau.edu.cn (M.L.); ymchen6426@163.com (Y.C.); fanweihzau@foxmail.com (W.F.); 18208986042@163.com (Z.L.); chenxu92721@163.com (X.C.); kw_tian@126.com (K.T.); 2 State Key Laboratory of Crop Stress Adaptation and Improvement, School of Life Sciences, Henan University, Kaifeng 475001, China; xiaohan@henu.edu.cn (H.X.); yaqipeng@163.com (Y.P.); zhangbao0535@163.com (B.Z.); 3 Arizona Genomics Institute, School of Plant Sciences, University of Arizona, Tucson, AZ 85721, USA; seunghl@ag.arizona.edu (S.L.); dkudrna@email.arizona.edu (D.K.); rwing@email.arizona.edu (R.A.W.); jzhang@mail.hzau.edu.cn (J.Z.); 4 National Key Laboratory of Crop Genetic Improvement, Huazhong Agricultural University, Wuhan 430070, China

**Keywords:** soybean, Iso-Seq, nodule development, differentially splicing event, major isoform switch, long non-coding RNA

## Abstract

Alternative splicing (AS) is a ubiquitous phenomenon among eukaryotic intron-containing genes, which greatly contributes to transcriptome and proteome diversity. Here we performed the isoform sequencing (Iso-Seq) of soybean underground tissues inoculated and uninoculated with Rhizobium and obtained 200,681 full-length transcripts covering 26,183 gene loci. It was found that 80.78% of the multi-exon loci produced more than one splicing variant. Comprehensive analysis of these identified 7874 differentially splicing events with highly diverse splicing patterns during nodule development, especially in defense and transport-related processes. We further profiled genes with differential isoform usage and revealed that 2008 multi-isoform loci underwent stage-specific or simultaneous major isoform switches after Rhizobium inoculation, indicating that AS is a vital way to regulate nodule development. Moreover, we took the lead in identifying 1563 high-confidence long non-coding RNAs (lncRNAs) in soybean, and 157 of them are differentially expressed during nodule development. Therefore, our study uncovers the landscape of AS during the soybean-*Rhizobium* interaction and provides systematic transcriptomic data for future study of multiple novel directions in soybean.

## 1. Introduction

Soybean (*Glycine max*), one of the most important crops in the world, is consumed as a major source of plant proteins and dietary oil [1]. As a legume, soybean can be infected by Rhizobium and harmoniously establish root nodule symbiosis with these bacteria to convert the atmospheric nitrogen into ammonia which can be utilized by plants [2]. Huge amounts of genes have been reported to be differentially expressed and regulated to control the rhizobia infection, nodule organogenesis or nitrogen fixation [3,4,5], through coordinating regulation in transcriptional, post-transcriptional or/and protein levels, like *Nodule Inception* (*NIN*) [6,7].

Alternative splicing (AS) is an extensive post-transcriptional regulatory mechanism, which plays a crucial role in diverse biological processes. Over 95% of human multi-exon genes [8] and 60% of intron-containing genes in plants [9] were found to undergo AS. In plants, plenty of research showed that AS plays a key role in embryogenesis [10], germination [11] and response to various external stimuli, such as heat [12], salt stress [13] and pathogen defense [14]. However, there are only several AS events reported during legume-rhizobia symbiosis. The *t-SNARE protein* (*MtSYP132*) [15,16], *phosphatidylinositol phospholipase C-like protein* (*MtDNF2*) [17], transcription factor *MtHAP2-1* [18] and *SymRK-interacting protein 1* (*LjSIP1*) [19] produced multiple isoforms with alternative termination, alternative donor, retained intron and skipped exon, respectively, which involved in bacteroid differentiation and nodule organogenesis. Thus, whether other nodulation-related genes regulated are by AS remains unknown.

Post-transcriptional splicing greatly contributes to proteome diversity as a result of complicated AS events [20]. There are five major classes of AS events, including exon skipping (ES), alternative 5′ splicing sites (A5SS), alternative 3′ splicing sites (A3SS), intron retention (IR) and mutually exclusive exon (MXE), which occurred diversely between plants and mammals [21,22]. Furthermore, studies on human cell lines revealed that about three-quarters of protein-coding genes could express two or more dominant isoforms with distinct AS events [23], and the relative abundance of transcripts may vary tremendously under different conditions in *Arabidopsis thaliana* [24]. Therefore, exploring the dynamic changes of different AS types during nodule development would provide an important basis for understanding the function of different isoforms in soybean interaction with rhizobia, nodule organogenesis and nitrogen fixation.

In recent years, lncRNAs have attracted increasing attention as a pivotal and complex regulator among many processes in eukaryotes [25,26]. LncRNAs are transcribed and spliced the same way as mRNAs, while lncRNAs tend to be shorter and express at lower levels [27]; they also do not have apparent coding potential [28]. Most studies found that lncRNAs as key regulators regulated gene expression in *cis* or *trans* [29]. In addition, they were involved in development [30], aging [31] and stress responses [32]. Although the functions of two lncRNAs from *trans-acting small interference RNA3* (*TAS3*) and antisense strand of *ENOD40* loci, have been demonstrated in legume-rhizobia symbiosis [33,34]. The global dissection of lncRNAs during nodule development is still limited.

Post-transcriptional regulation as a pervasive regulatory way may play an indispensable role in nodule development. In this study, to elucidate the comprehensive landscapes of AS and lncRNA during nodulation, we combined the PacBio Iso-Seq and Illumina RNA-Seq applications to obtain the full-length transcriptome of soybean underground tissues inoculated and uninoculated with *Bradyrhizobium japonicum* USDA110. In total, 200,681 full-length transcripts with high quality from 26,183 loci were identified from Iso-Seq analysis. To explore the splicing dynamic during nodule development, over 7000 differentially AS events were detected between inoculated and uninoculated samples, which showed obvious temporal response patterns. Interestingly, there were more than 2000 loci with differential transcript usages during soybean symbiosis. Moreover, we identified the differentially expressed lncRNAs and they are specifically and highly expressed at late stages, which may play an important role in nitrogen fixation. Overall, our findings provide a valuable resource for understanding the dynamic regulation of nodulation at the post-transcriptional level.

## 2. Results

### 2.1. Genome-Wide Characterization of Full-Length Transcripts in Soybean by Iso-Seq

To understand transcriptome diversity in soybean nodulation at different developmental stages, we performed both PacBio Iso-Seq and Illumina RNA-Seq for soybean nodules after inoculation and corresponding root parts without Rhizobium infection. For RNA-Seq, 54 individual RNA samples at nine time points, including 1, 4, 6, 8, 10, 15, 20, 25 and 30 dpi, were extracted with three replicates. For Iso-Seq, RNAs from the nine time points were mixed into inoculated and uninoculated groups, then sequenced separately (Section 4 and Figure S1). We followed a universal computational workflow for analyzing Iso-Seq and RNA-Seq data (Figure S2), including separate data processing, annotation, and classification of Iso-Seq isoforms, and combinative analysis of AS and transcript-level expression.

Twenty-six SMRT cells for five fraction libraries (1–2 kb, 2–4 kb, 3–5 kb, 3.5–6 kb and >4 kb) were applied to perform Iso-Seq for each RNA sample, thus data from 52 SMRT cells in total were collected in this study (Table 1). We obtained 4,890,693 polymerase reads, and 1,983,093 ReadofInserts were classified as FLNC transcripts. After reads clustering and error correction, 452,703 high-quality isoform sequences with accuracy > 98% were used for the following analyses. Among them, 99.55% of the high-quality isoforms were able to be mapped to the *Glycine max* cv. Jidou 17 (JD17) reference genome, and 200,681 unique transcripts remained after removing redundancies.

**Figure 1:**
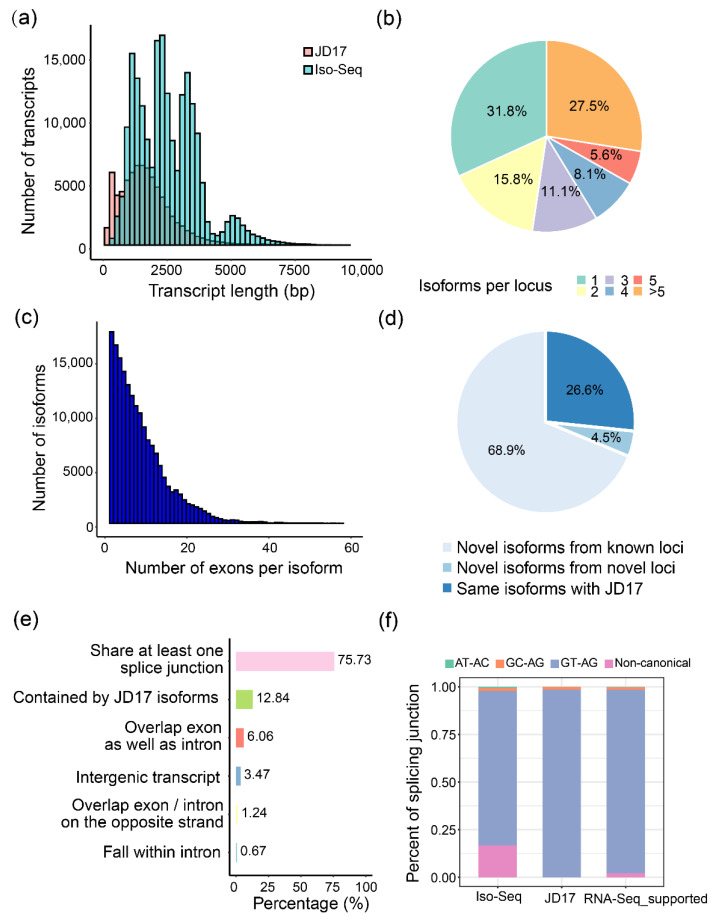
Summary of the Iso-Seq data. (**a**) The distribution of transcripts length for *Glycine max* cv. Jidou 17 (JD17) reference (pink) and Iso-Seq data (green); (**b**) Pie chart for the percentage of isoform numbers per locus; (**c**) Number of exons per transcript for the Iso-Seq transcripts; (**d**,**e**) Composition of the Iso-Seq isoforms relative to the JD17 reference (**d**) and detailed categories of novel isoforms compared to the JD17 reference (**e**); (**f**) Splicing junctions of the Iso-Seq data versus the JD17 reference.

Length distribution comparison of the non-redundant transcripts (average of 2618 bp per transcript) with the transcripts annotated in the JD17 (average of 1759 bp per transcript) indicated that the Iso-Seq generated much longer isoform sequences, and more full-length transcripts were captured in this study (Figure 1a). In total, 200,681 unique transcripts are richly distributed in the pericentric regions on all 20 chromosomes (Figure S3) and cover 26,183 different loci. Above 65% of these loci produced more than one isoform, similar to that in *Arabidopsis thaliana* [9] (Figure 1b). About 66.78% of the multi-isoform loci produced two to six isoforms (Figure 1b and Figure S4). Variation in exon number per gene is enormous among eukaryotic genomes [35]. We also found, on average, there are 8 exons per isoform by calculating the exon number for PacBio transcripts (Figure 1c). Among these isoforms, 11.05% of them were of single exons, and the others were formed from multiple exons. Furthermore, 97.23% of these multiple-exon isoforms have AS variants.

About 70% (138,235) of the identified transcripts are novel isoforms from the known annotated loci, and 9043 transcripts are from novel loci (Figure 1d,e). These novel isoforms were classified into eight groups according to their original loci relative to the JD17 transcripts, and 75.73% of them share at least one splicing junction with the annotated transcripts, indicating that post-transcriptional AS contributes greatly to the transcriptome diversity. We randomly selected thirteen isoforms from five loci and confirmed their existence by RT-PCR assays (Figure S5).

To mutually confirm RNA-Seq and Iso-Seq transcripts, the reference-guided assembly was performed on short reads, and transcripts from Iso-Seq and short reads-assembled were quantified (Figure S6a). We found that the Iso-Seq data could cover more than 80% of the RNA-Seq-covered loci and JD17 reference genes, especially for those loci with Fragments Per Kilobase per Million mapped reads (FPKM) > 0.1 (Figure S6b). There are more isoforms detected by Iso-Seq than by assembled transcripts (Iso-seq: 101,807 vs. RNA-seq assembly: 39,231, FPKM > 0.1), which suggests that Iso-Seq have a higher sensitivity to identifying isoform variants. In addition, although the majority of gene loci (96.17%) in Iso-seq can be identified by RNA-Seq, less than 30% of the Iso-Seq transcripts can be accurately assembled by RNA-seq (Figure S6b, FPKM > 0.1), indicated the short reads assembly have a high false-positive rate and it is difficult to identify complete isoform structures. Based on the distribution of genes/isoforms’ FPKM value, genes with FPKM > 0.1 and isoforms with FPKM > 0.01 were applied as the threshold throughout this study to cover more biologically meaningful transcripts (Figure S6a).

### 2.2. Comprehensive Temporal Analysis of Alternative Splicing Dynamics during Nodule and Root Development

Iso-Seq can capture full-length isoforms and resolve their complete structures without assembly, which makes it the best choice to profile AS. We identified 82,259 AS events in soybean that consists of 19,307 types of splicing junction structures from the Iso-Seq data. Although only covering 26,183 loci, the Iso-Seq data was still disclosed tremendously more AS events and types in soybean roots and nodules than the JD17 reference annotation (17,258 events from 880 types) that cover over 50,000 loci (Figure S7). The top ten AS forms from the Iso-Seq data and their occurrence (including four common types) are shown in Figure S8.

We mapped short reads to the genome to further evaluate the ability and accuracy of Iso-Seq detecting AS events and found that 82.16% of splicing junctions in the Iso-Seq transcripts are supported by the RNA-Seq reads (Figure 1f). Thus, without the high false-positive rates of assembling isoforms from short reads (Figure S6b), the Iso-Seq approach accurately identified most AS events.

**Figure 2:**
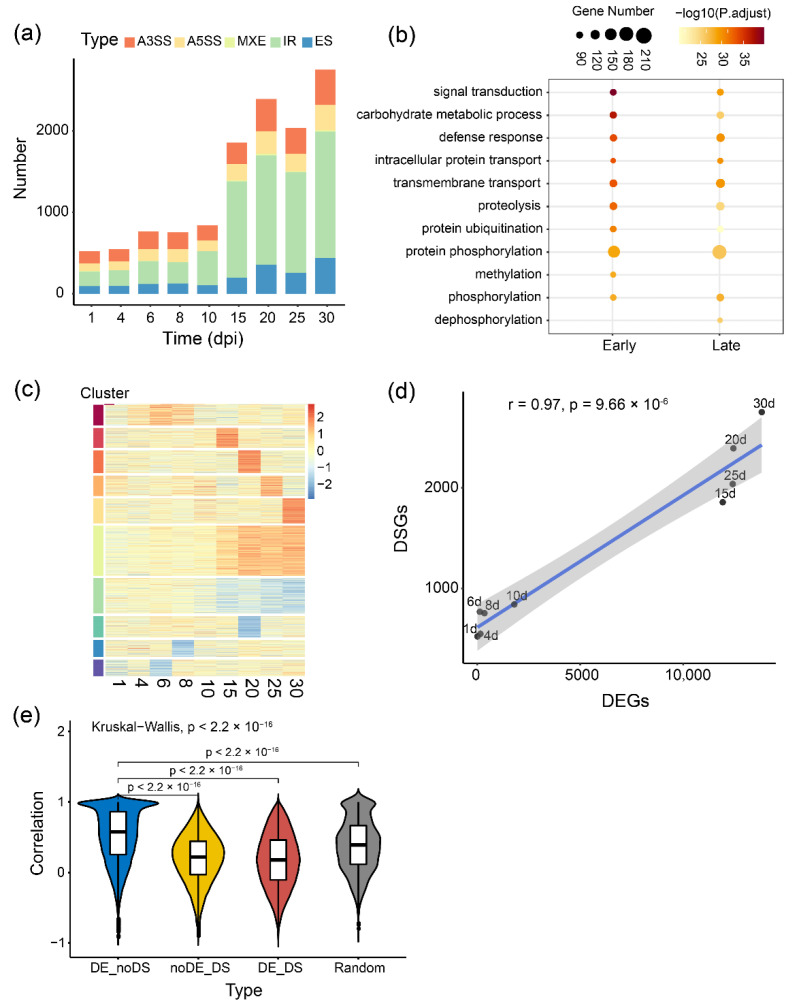
Genome-wide analysis of alternative splicing dynamics during nodule development. (**a**) Number of differentially splicing events at each time point. Different colors represent five types of common AS events. IR: Intron retention, A3SS: Alternative 3′ splicing site, A5SS: Alternative 5′ splicing site, ES: Exon skipping, MXE: Mutually exclusive exons. dpi: day post-inoculation; (**b**) GO enrichment analysis of the differentially spliced genes at early stage (Early, 1 to 10 dpi) and late stage (Late, 15 to 30 dpi); (**c**) Heatmap of the global alternative splicing changes measured by ∆PSI between inoculated and uninoculated groups over time courses. Red represents higher PSI under inoculated conditions, and blue represents the lower. ∆PSI: The difference of percent spliced in; (**d**) The number of the differentially spliced genes (DSGs) versus the differentially expressed genes (DEGs) at 9 time points. Pearson correlation coefficient (PCC) was performed, *p*-value = 9.66 × 10^−6^; (**e**) Spearman correlation coefficient (SCC) of the expression between transcripts and their corresponding genes. 16,301 DE but not DS genes (DE_noDS, N = 71,604, r = 0.52), 630 DS but not DE genes (noDE_DS, N = 20,503, r = 0.20), 1888 DS and DE genes (DE_DS, N = 77,884, r = 0.17) and 1000 random selected genes (Random, N = 4976, median SCC = 0.39). Kruskal–Wallis test was used, *p* < 2.2 × 10^−16^. N showed the number of genes-transcripts pairs for correlation calculation.

Furthermore, to understand whether and how AS is regulated during symbiotic nodulation, we applied the RNA-Seq short reads to quantify AS events and conducted differential transcript splicing analysis during nodule development. Five typical AS changes, including A3SS, IR, ES, A5SS and MXE, were identified between inoculated and uninoculated groups at each time point. After filtering with criteria FDR < 0.05 and percent spliced in difference (∆PSI) > 0.1, 7874 differentially splicing events from 2518 loci were identified. The number of differentially AS was dramatically increased at 15 dpi (Figure 2a). The differentially spliced genes at early stages (before 15 dpi) and late stages (at and after 15 dpi) of nodule development were identified (1401 and 1969 genes), and further gene ontology (GO) enrichment analysis of these genes highlighted certain specific biological processes (Figure 2b). For example, ‘signal transduction’, ‘carbohydrate metabolic process’, ‘defense response’, ‘ubiquitination and phosphorylation’ and ‘transport’, are persistently regulated at post-transcriptional levels through entire nodule development. These findings suggested that some loci involved in plant-bacteria interaction, nodule initiation and growth, and nitrogen fixation undergo significant AS, which adds another layer of coordinate regulation of the symbiosis between soybean and *Rhizobium*.

Further global splicing analysis at different time points of nodule development revealed the dynamic AS changes and 10 distinct temporal patterns of AS during this process (Figure 2c). Interestingly, different AS types displayed some specific patterns (Figure S9). For example, IR (Cluster 3 and 4) and ES (Cluster 8 and 9) events all showed gradual changes after 15 dpi, whereas most of the splicing changes for A5SS and MXE were stage-specific (Figure S9). These results implied that different types of AS events might be regulated by different splicing factors at different developmental stages, especially at the late stage of nodule development. Two differentially spliced events were randomly selected and confirmed by RT-PCR (Figure S10).

As a vital way of post-transcription regulation, alternative splicing might be associated with the level of gene expression [36,37]. Using IR as an example, we found 3431 differentially spliced (DS) exons from 1000 genes, 85.3% of which were also differentially expressed (DE). We further tested whether the expression of certain genes involved in nodulation or nitrogen fixation is coordinated with splicing signal and transcript abundance. Our analysis of the number of DS events versus DE genes showed a significant linear correlation across nine time points (Figure 2d, r = 0.91, *p*-value = 9.66 × 10^−6^), indicating the consistency of AS with gene expression at the whole transcriptome level. To further investigate the relationship between expression and AS, we calculated spearman correlation coefficients (SCC) of the expression level between 16,301 DE but not DS genes (DE_noDS) and their corresponding transcripts, 630 DS but not DE genes (noDE_DS) and their corresponding transcripts, as well as 1888 DS and DE genes (DE_DS) and their corresponding transcripts, respectively. We found that the median SCC of DE_noDS genes (r = 0.52) is dramatically higher than noDE_DS genes (r = 0.20) and DE_DS genes (r = 0.17, Figure 2e, *p*-value < 2.2 × 10^−16^). The above results suggested that transcription and splicing might differentially influence certain isoform expressions to participate in nodulation.

### 2.3. Differential Transcript Usage Evaluated by Isoform Expression Levels

**Figure 3:**
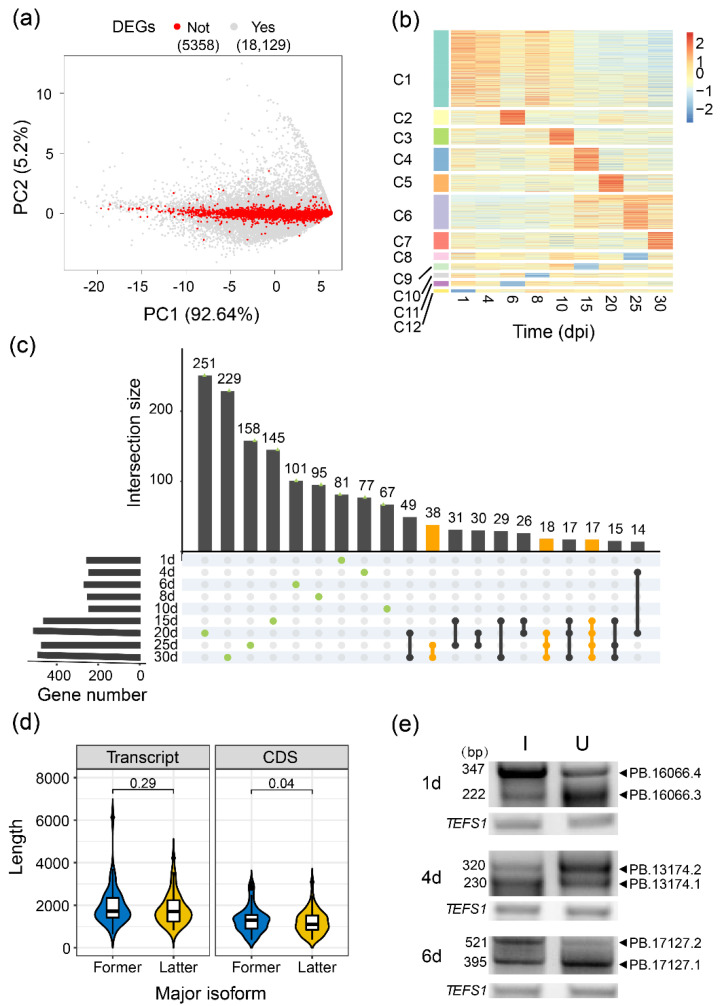
Global differential transcript usage analysis on isoform level. (**a**) PCA analysis of all expressed genes. Red points showed the not differentially expressed genes (nDEGs), grey points showed the differentially expressed genes (DEGs); (**b**) Expression patterns of differentially expressed transcripts (DETs) from nDEGs. The values were calculated by (FPKM _Inoculation_ + 1)/(FPKM _Uninoculation_ + 1) and scaled using Z-score; (**c**) Intersections of various gene sets experienced major isoform switches between inoculated and uninoculated conditions during nodule development, including stage-specific (green dots) and continuous changes (yellow lines); (**d**) Comparison of transcript and CDS lengths between the former and latter isoforms, where major isoform switched irreversibly and characteristically under inoculation; (**e**) RT-PCR validation of expression levels DETs from (**b**).

The expression level and stability of an mRNA largely determine the protein abundance. However, because limited types of the identified AS events and exons are shared by multiple isoforms, the differentially exon-centric splicing does not definitely give rise to the relative changes in transcript expression. To discover AS regardless of gene transcription regulation, we analyzed transcript expression alterations for the isoforms that were significantly differentially expressed at least at one time point, but their corresponding genes did not show a significant difference between the inoculated and uninoculated samples (Figure 3a). We obtained 9448 differentially expressed transcripts from 2101 not differentially expressed genes (39.21%), which were classified into 12 expression clusters representing different temporal expression patterns across all developmental stages (Figure 3b). Further GO enrichment analysis for each cluster indicated that these transcripts from different patterns participated in ‘RNA processing’, ‘transport’, ‘glucan biosynthetic process’ and ‘gene silencing by RNA’. Interestingly, ‘DNA repair’ and ‘base-excision repair’ were induced, especially after 15 dpi, whereas ‘photosynthesis’, ‘fatty acid and trehalose biosynthetic processes’ and ‘carbohydrate metabolic process’ were inhibited after inoculation (Figure S11). These results suggested that the difference in transcripts expression caused by AS plays an important role in regulating soybean nodule development. Three random differentially expressed transcripts from different time points were detected using RT-PCR (Figure 3e).

To systematically analyze the functional consequence of AS by a transcript-centric approach, we calculated the expression ratio of each isoform to its corresponding gene. Based on the relative abundance of each isoform, we defined the isoforms with an FPKM of more than 50% of all isoforms’ FPKMs on the same locus as a major isoform under each condition of each time point. Among all 24,001 genes with 193,324 expressed transcripts (FPKM > 0.01 at least in one sample) analyzed, 86.18% of them contain one major isoform (30.88% of them produce only one expressed isoform) or are without a major isoform, which was determined as genes without major isoform switch during nodule and root development. Importantly, we also found 3315 genes producing distinct major isoforms under different conditions. As the ratio change of most isoforms’ relative expression was relatively small (Figure S12, median = 0.3), we excluded the major isoforms switches caused by diminutive difference (30% as a cutoff). The remaining 2008 genes undergoing obvious major isoform switches between inoculated and uninoculated conditions during development were further analyzed (Figure 3c). Most isoform switches were transient and happened only at one stage (green dots), but 73 genes underwent continuous changes in transcripts forms from 15/20/25 dpi to 30 dpi (yellow lines and dots). We further found that 51 of these genes shifted their major isoforms from the former into the latter structures in the inoculated condition but kept the former structures under the uninoculated conditions. Predicted CDS length of 51 genes was significantly changed between their two major isoforms (Figure 3d, Wilcoxon test, *p*-value < 0.05), about one-fifth of which showed domains difference between the former and latter dominant isoforms (Table S2).

One example of significant isoform switches happened at 15 dpi in *PB.12184* locus, and it has no corresponding gene in JD17 annotation (Figure S13a). This locus encodes a disease resistance protein based on homologous genes annotation (*Glyma.18G117800* in *Glycine max* cv. Williams 82 (Wm82.a2.v1) and *AT5G17680* in *Arabidopsis thaliana*), which belongs to TIR-NBS-LRR family. A longer isoform with intact structure (*PB.12184.1*) was induced after inoculation and down-regulated in uninoculated conditions at 15 dpi, suggesting that defense response was also induced at the nitrogen-fixation stage of nodule compared to root development. Another locus *PB.18879* (*JD004G0127700* in JD17) exhibits different transcripts usage at 20 dpi between two conditions, encoding the *response regulator 9* (*ARR9*, Figure S13b). One of the isoforms (*PB.18879.2*) with an extra transmembrane region was highly expressed in an uninoculated sample, implying that ARR9 may have different protein localization during root development at 20 dpi. Moreover, the *PB.17146* locus (ankyrin repeat protein, *AT2G45360* as its orthologous gene) with alternative untranslated regions (UTR) had undergone a major isoform switch at 30 dpi (Figure S13c), which may affect mRNA stability, localization and translation efficiency [38,39] at the nitrogen-fixation stage.

### 2.4. Long Non-Coding RNA Identified from Iso-Seq Transcripts

**Figure 4:**
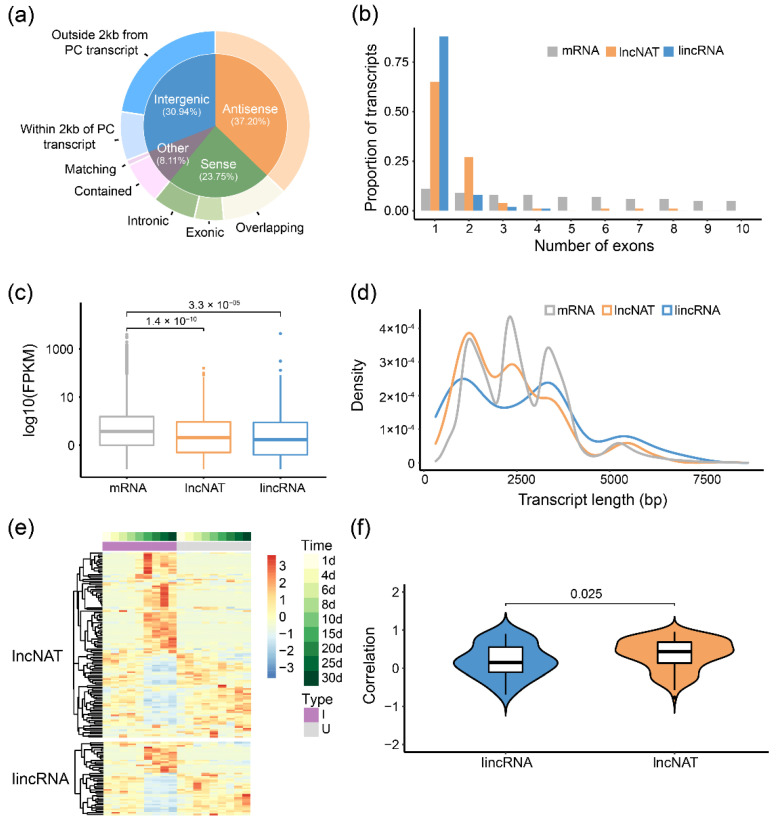
Characteristics of long non-coding RNAs in soybean root and nodule tissues. (**a**) Classification of lncRNAs according to their intersection with JD17-annotated protein-coding transcripts on the genome. There are four part of these categories and eight subcategories; (**b**–**d**) lincRNAs and lncNATs were compared to Iso-Seq-detected mRNA on (**b**) exon numbers per transcript, (**c**) expression levels and (**d**) length distributions. Grey stands for mRNA, red for lncNATs (antisense lncRNAs), blue for lincRNAs (intergenic lncRNAs without 2 kb of protein-coding transcripts). Wilcoxon test was used; (**e**) Expression patterns of 157 differentially expressed lincRNAs and lncNATs; (**f**) The spearman correlation coefficient (SCC) of expression pattern between differentially expressed (DE) lncRNA and corresponding protein-coding (PC) transcripts (lncNATs with anti-strand PC transcripts and lincRNA with adjacent PC transcripts) across the root and nodule development. Wilcoxon test was used.

LncRNAs were involved in many biological processes in both plants and animals, but the investigation of the whole-genome lncRNAs during soybean-rhizobia symbiosis is lacking. To identify lncRNAs potentially involved in soybean nodulation, we predicted 2634 non-coding RNAs from all Iso-Seq isoforms based on a model built with *Arabidopsis thaliana* lncRNA. After excluding transcripts with protein-coding potential (Methods), 1563 high-confidence lncRNAs were identified and used for subsequent analysis. According to their biogenesis on genome location relative to JD17-annotated protein-coding (PC) transcripts, we divided 1563 lncRNAs into four categories and eight subcategories (Figure 4a and Figure S14). Long non-coding intergenic RNAs (lincRNAs, 22.63%) referred to outside 2 kb from a PC transcript and long non-coding nature antisense transcripts (lncNATs, 37.20%), accounting for great proportions of lncRNAs, were used for comparison with protein-coding transcripts. We found that lincRNAs and lncNATs tend to have fewer exons per transcript and show lower expression levels, but they are not obviously shorter than mRNAs in length (Figure 4b–d).

Among the total 907 lincRNAs and lncNATs, 157 of them were found differentially expressed between the inoculated and uninoculated conditions at least at one time point. We further analyzed the correlation between differentially expressed lncRNAs and their anti-strand (for lncNATs) or adjacent (for lincRNAs) PC transcripts and found that most of these pairs are positively related (Figure 4f). Moreover, the correlation between lncNATs and their anti-strand PC transcripts was significantly higher than lincRNAs (Median r: 0.43 vs. 0.14, Figure 4f), suggesting that lncNATs may mainly affect the expression of anti-strand PC transcripts to regulate nodule development. Five differentially expressed lincRNAs/lncNATs were validated by RT-PCR (Figure S15).

## 3. Discussion

In this study, we explored complicated post-transcriptional regulation during soybean nodulation by Iso-Seq. First, we found that transcripts from soybean underground tissues are greatly longer and more abundant than the annotated transcripts of soybean JD17 genome [40] (Annotation with PacBio Iso-Seq datasets from *Glycine max* cv. Williams 82 mixed tissue [41]), probably because of deeper sequencing depth (52 vs. 23 SMRT cells) and longer library length. Second, we identified nearly 8000 differentially splicing events after inoculation and obtained dynamic AS profiles. Moreover, thousands of genes with major isoforms switches between different conditions were discovered and analyzed, which provides novel evidence for the AS-influenced nodule formation and development in soybean. Finally, by identifying poly(A)+ full-length lncRNAs from Iso-Seq data, we spotted that more than a hundred of lncRNAs were differentially expressed and the expression between lncNATs and their anti-strand PC transcripts was highly correlated, suggesting that lncRNAs might be another post-transcriptional level to regulate gene expression during nodule development.

The large number of differential AS events involved in nodule development uncovered that post-transcriptional regulation plays a crucial role in the symbiosis between soybean and *Rhizobium*. Previous studies revealed that AS exists widely among different tissues and developmental stages in soybean [37]. From Iso-Seq data, we identified that 80.78% of multi-exon loci produced more than one isoform in soybean roots and nodules, suggesting the vital function of AS events in symbiosis. Interestingly, many orthologous genes which are necessary for nodulation produced many splicing variants. For instance, two soybean orthologous genes of the *Lotus histidine kinase 1* (*LjLHK1*) [42]/*Medicago cytokinin response 1* (*MtCRE1*) [43] gene are highly alternatively spliced (32 isoforms from *GmCRE1a* and 58 isoforms from *GmCRE1b*). It is well-studied that *MtCRE1*/*LjLHK1* was involved in various processes during legume symbiosis after perceiving cytokinin, such as activating cortical division in nodule initiation [44,45], promoting autoregulation of nodulation (AON) pathway for restricting excessive infection [46,47]. Remarkably, we found that some of these distinct *GmCRE1a/b* isoforms show different expression patterns across nine time points during nodule and root development (Figure S16), suggesting that *GmCRE1a/b* are regulated at both transcriptional and post-transcriptional levels during symbiosis. Therefore, different isoforms of these genes may have specific functions to expand functional and phenotypic diversity for coordinating nodule development. The functional exploration of novel and vital isoforms in nodulation will greatly accelerate the precise molecular design and breeding for legumes in the future [48,49].

LncRNAs may have diverse features and regulatory mechanisms in soybean nodule development. Unlike previous studies in humans and plants [27,50], lncRNAs identified from our Iso-Seq data in soybean are not significantly shorter than mRNAs (Figure 4d), which may be due to the exclusion of most short RNAs (<1 kb) by following Iso-Seq size selection protocol. Another possible reason is that the length of lncRNAs could have been previously underestimated as a result of low expression thus making it difficult for short reads assembly, which was hinted at by comparing novel lncRNAs from Iso-Seq to the known assembled lncRNAs in maize [51].

In addition, lincRNAs are distinguished from mRNA in the transcriptional and splicing regulation in humans [52], suggesting that lncRNAs may also exhibit different AS dynamic changes. It was also reported that lncRNAs can modulate AS patterns by hijacking nuclear AS regulators [53] and lncNAT can form RNA-RNA duplexes with sense pre-mRNA to inhibit splicing [54]. Hence, our data provide abundant and precise resources for studying the relationship between AS and lncRNA during nodulation.

Soybean-*Rhizobium* symbiosis, as the result of numerous environmental stimuli and developmental cues, may cause the occurrence of erroneous transcripts, which could be recognized and degraded by the nonsense-mediated decay (NMD) pathway, to maintain transcriptome stability [55,56]. There are several premature termination codons (PTCs) features about NMD-responsive transcripts, including long 3′ UTR (>580 nt, greater than the third quartile of all 3′ UTR length, Figure S17) and splicing junctions within 3′ UTRs, along with the length between termination codon and the last exon-exon splicing junction longer than 50 nt [55,56]. We preliminarily identified that 25,083 isoforms (13.66% of isoforms containing 3′UTR) harbor those typical NMD features, consistent with that in *Arabidopsis thaliana* [57]. Moreover, it has been reported that upstream ORF (uORF) also could trigger NMD [58]. Therefore, further investigation of these transcripts with uORFs and PTCs is of great interest to understand post-transcriptional NMD regulation during nodule development.

## 4. Materials and Methods

### 4.1. Materials and Growth Conditions

Soybean seeds (*Glycine max* cv. Jidou 17) were sterilized with chlorine gas (5 mL of 32% (*w*/*w*) HCl (Sigma Aldrich, Saint Louis, MO, USA) to 100 mL 4–5% (wt/vol) sodium hypochlorite (Sigma Aldrich, Saint Louis, MO, USA) in a beaker) for 15 h [59] and then left in a sterilization hood for 2 h. The sterilized seeds were sown in growing bottles filled with sand after soaking in sterile Milli-Q water (Milli-Q, ELGA Veolia, United Kingdom) for 30 min and watered with sterile Fahraeus solution [60] containing 2 mM KNO3 (Sigma Aldrich, Saint Louis, MO, USA). Seeds grew in a growth chamber with 60% humidity under 8 h–16 h light–dark conditions at 28 °C and 23 °C. *Bradyrhizobium japonicum* USDA110 was used in our study, which grew in HEPES (Sigma Aldrich, Saint Louis, MO, USA), and MES (Sigma Aldrich, Saint Louis, MO, USA) buffered medium [61] with 20 mg/L chloramphenicol (Sigma Aldrich, Saint Louis, MO, USA) at 28 °C for 3 d. Rhizobia were collected and diluted with sterile water to OD600 = 0.1. Seedlings (aged 10 d) were inoculated with 1ml rhizobia solution or sterile Milli-Q water.

### 4.2. RNA Preparation

Underground tissues of soybean were collected at 1, 4, 6, 8, 10, 15, 20, 25 and 30 days post-inoculation (dpi, Figure S1). Roots or nodules from five plants were pooled at each time point. Total RNA was extracted with TRIzol reagent (Invitrogen 15596026, Thermo Fisher Scientific, Waltham, MA, USA). Nine RNA samples inoculated with *Bradyrhizobium japonicum* USDA110 and water were equally mixed for the inoculated and uninoculated samples, respectively. Then, the inoculated and uninoculated samples were separately used for Iso-Seq.

### 4.3. Library Preparation and Sequencing

For Illumina RNA-Seq, one microgram of the 54 individual RNA samples with 3 replicates were obtained. RNA of each sample was qualified and used to construct next-generation sequencing libraries (Strand-Specific) by Novogene company (Beijing, China). Finally, 150 bp paired-end reads were generated from Illumina HiSeq 4000 platform. For PacBio Iso-Seq, one microgram of each mixed RNA sample was prepared to construct Iso-Seq libraries. The cDNAs of each sample were synthesized by Clontech SMARTer PCR cDNA synthesis Kit (cat. no.634926, TaKaRa, Shiga, Japan, Avaliable online: http://www.clontech.com/, accessed on 4 June 2022) and the quality control of products was implemented with a 2100 BioAnalyzer (Agilent, Santa Clara, CA, USA). According to the size fractions, five libraries with different fragment lengths (1–2, 2–4, 3–5, 3.5–6 and 4–10 kb) were generated for each sample by a Sage BluePippin size selection system (Sage Science, Beverly, MA, USA, Available online: http://www.sagescience.com/, accessed on 4 June 2022) and the SMRTbell libraries were constructed through Pacific Biosciences SMRTbell Template Prep Kit 1.0 (PacBio, Menlo Park, CA, USA, Available online: http://www.pacb.com/, accessed on 4 June 2022).

All of the libraries were sequenced by PacBio RS II real-time sequencer. The libraries of 1–2, 2–4 and 3–5 kb range were sequenced with 6 Single-Molecule Real-Time (SMRT) cells for each, and the 3.5–6 and 4–10 kb libraries with 4 SMRT cells for each. A total of 26 SMRT cells were used for each sample (Table 1).

### 4.4. RT-PCR for Validation

RNAs from different nodule developmental stages for Iso-Seq were used for PCR validation. cDNAs were synthesized by reverse transcriptions with Reverse Transcriptase M-MLV (RNase H-) (cat. no.639575, TaKaRa, Shiga, Japan, http://www.takarabiomed.com.cn, accessed on 4 June 2022). Two micrograms total of RNA was used for the synthesis of the first-strand cDNA, and then the validation of different isoforms and lncRNAs from Pacbio sequences was carried out. PCR amplification was performed with the designed specific primers by 2 × Taq Plus Master Mix (Dye Plus) (P212-01, Vazyme, Nanjing, China, http://www.vazyme.com, accessed on 4 June 2022). *Soybean elongation factor 1-alpha* (*TEFS1* gene, *JD017G0182700*) was used as the control. All specific primers used for PCR were listed in Table S2. For each RT-PCR experiment, three biological replicates were conducted.

### 4.5. Pacbio Data Analysis

The Pacbio reads were processed by RS_Iso-Seq 2.3, the SMRT Iso-Seq analysis protocol. Each raw polymerase read was extracted from one ReadOfInsert measured by the number of full passes > 0 and predicted consensus accuracy > 0.75. Next, ReadsOfInsert was classified as full-length non-chimeric reads (FLNCs) and non-full-length reads. After the isoform level cluster, high-quality (accuracy > 98%) consensus reads were obtained. We gathered high-quality isoforms from two samples and different size libraries together and mapped them to the *Glycine max* cv. Jidou 17 (JD17) reference genome [40] with GMAP [62] with parameter -n 2. Collapse_isoforms_by_sam.py (Python-3.8.0) in pbtranscript-tofu was applied to remove redundant isoforms with the parameters: min-coverage = 0.85, min-identity = 0.9 and --dun-merge-5-shorter (https://github.com/PacificBiosciences/cDNA_primer/wiki, accessed on 4 June 2022). The generated gff file was used for detecting AS events by Astalavista v4.0 [63] on the ‘complete’ mode and following analysis as Iso-Seq annotation reference (Iso-Seq gff).

### 4.6. Integrative Analysis of Iso-Seq with Illumina Short Reads

After quality control with Trimmomatic v0.27 [64] to remove low-quality reads, the RNA-Seq short reads were mapped to JD17 reference genome by HISAT v2.1.0 [65] with Iso-Seq gff. Genes were quantified by Stringtie v2.1.5 [66] based on unique mapped reads and FPKM values were used for the normalization of gene expression. Differentially expressed genes and transcripts were identified by DESeq2 (R 3.5.0) and filtered using padj < 0.05. Cluster analyses were conducted by pheatmap (R 3.6.2) with ‘Euclidean’ and ‘average’ methods.

For comparing RNA-Seq assembled transcripts with Iso-Seq transcripts, short reads from RNA-seq were also mapped to the JD17 reference genome with the JD17 annotation file. Assembly and quantification of transcripts were conducted by Stringtie v2.1.5 [66] with default parameters. To identify the corresponding transcripts between Iso-Seq and RNA-Seq assembled transcripts, Cuffcompare v2.2.1 [67] with default parameters was used. To perform differential AS analysis, we used STAR v2.7.2 [68] and rMATS v4.0.2 [69] to estimate splicing changes from trimmed 125 bp short reads and Iso-Seq gff.

### 4.7. Annotation of Pacbio Isoforms

To completely predict open reading frames (ORFs) for all Iso-Seq isoforms (including those with unknown domains), TransDecoder v3.0.1 [70] was used to obtain possible coding sequence regions and retained the single best ORF for each of them. Based on Iso-Seq gff, we extracted corresponding nucleotide sequences for each isoform and performed transcript-level annotation by Interproscan5-RC6 [71]. Gene Ontology terms for all genes were annotated by Blast2GO [72]. Protein sequences were aligned to nr databases (updated on 31 January 2017) by BLASTP (2.2.28+). Subsequent enrichment analysis was performed with clusterProfiler [73]. Predictions of protein domain were conducted by the SMART database [74].

### 4.8. LncRNAs Identification

A training model was built by PLEK v1.2 [75] based on *Arabidopsis thaliana* annotated lncRNAs from Araport11. Soybean lncRNAs were predicted from all Pacbio isoforms using this model and filtered with ORFs > 100 aa (predicted by Transdecoder v3.0.1). To eliminate the transcripts similar to known protein-coding genes, the lncRNAs aligned to the proteins of *Arabidopsis thaliana,* rice and soybean from the Swiss-Prot database were removed (BLASTX v2.2.28+ [76] with E-value ≤ 1 × 10^−10^).

## Figures and Tables

**Table 1:** Summary statistics of Iso-Seq data.

Library Size (kb)	SMRT Cells	Polymerase Reads	FLNC ^1^	High Quality Isoforms
1–2	12	905,500	419,736	117,970
2–4	12	1,231,202	612,254	144,968
3–5	12	1,238,704	542,507	105,423
3.5–6	8	697,016	161,752	55,532
4–10	8	818,271	246,844	28,810
Total	52	4,890,693	1,983,093	452,703

^1^ FLNC: full-length non-chimeric reads.

## Data Availability

The raw data described in this paper have been deposited at NCBI Sequence Read Archive (SRP121077 and PRJNA852352).

## Supplementary Materials

The following supporting information can be downloaded at: www.mdpi.com/article/10.3390/ijms23137371/s1.
