# Supplementary material for: Full-Length Transcriptome Sequencing Reveals Alternative Splicing and lncRNA Regulation during Nodule Development in Glycine max
Source: Int J Mol Sci. 2022 Jul 1;23(13):7371. doi: 10.3390/ijms23137371 (PMC9266934; doi:10.3390/ijms23137371)
Supplement: Supplementary file 1 [file ijms-23-07371-s001.zip › Supplementary figure.pdf]

Supplementary Information

Full-length transcriptome sequencing reveals alternative splicing and lncRNA regulation during nodule development in *Glycine max*

Jing Liu[1,2], Shengcai Chen[1,2], Min Liu[1], Yimian Chen[1,2], Wei Fan[1,2], Seunghee Lee[3], Han Xiao[2], Dave Kudrna[3], Zixin Li[1], Xu Chen[1,2], Yaqi Peng[2], Kewei Tian[1,2], Bao Zhang[2], Rod A. Wing[3], Jianwei Zhang[3,4], Xuelu Wang[2,*]

[1] National Key Laboratory of Crop Genetic Improvement, College of Life Science and Technology, Huazhong Agricultural University, Wuhan 430070, China.

[2] State Key Laboratory of Crop Stress Adaptation and Improvement, School of Life Sciences, Henan University, Kaifeng 475001, China.

[3] Arizona Genomics Institute and BIO5 Institute, School of Plant Sciences, University of Arizona, Tucson, AZ 85721, USA.

[4] National Key Laboratory of Crop Genetic Improvement, Huazhong Agricultural University, Wuhan, 430070, China.

[*] Correspondence: xueluw@henu.edu.cn

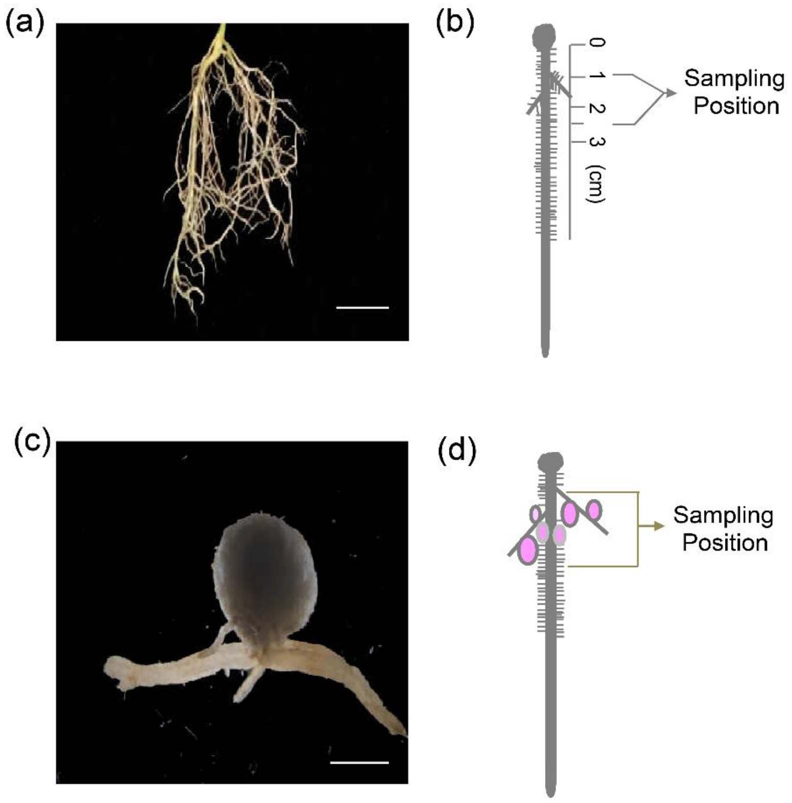

Figure S1. The schematic diagram of underground tissue used for Iso-Seq and RNA-Seq in soybean. **(a and b)** Collected tissues before nodule emerging; (**a**) The root image of soybean at 1 dpi (day post inoculation), bar = 2 cm; (**b**) The sampling position diagram of 1, 4 and 6 dpi. About 1 to 2.5 cm under the root and stem junction were sampled; (**c** and **d)** Collected tissues after nodule emerging; (**c**) The root and nodule image of soybean at 30 dpi, bar = 1 mm; (**d**) The sampling position diagram at 8, 10, 15, 20, 25 and 30 dpi. Nodules with adjacent roots were sampled.

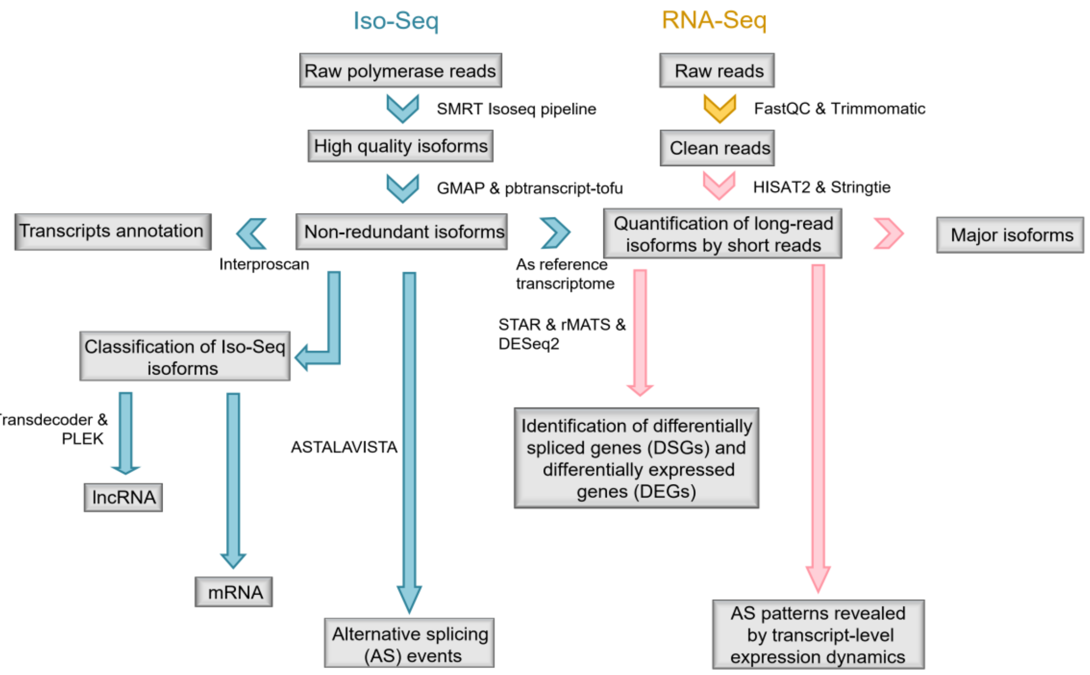

Figure S2. Flowchart of bioinformatic analysis for Iso-Seq and RNA-Seq. Green, yellow and pink arrows show the analysis using Iso-Seq, RNA-Seq and combined datasets, respectively.

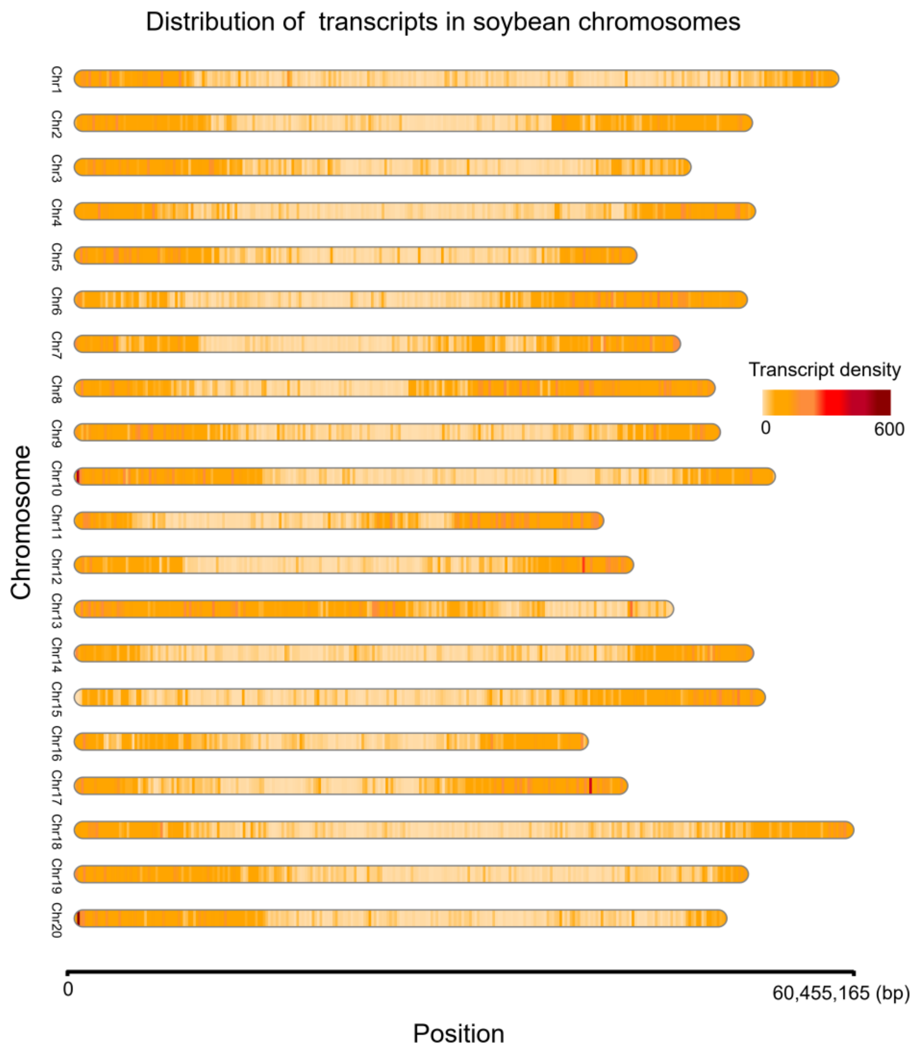

Figure S3. Distribution of all non-redundant transcripts along the 20 soybean chromosomes. Position of 200,681 transcripts along 20 soybean chromosomes, the color show density of transcripts for each 200 kb window.

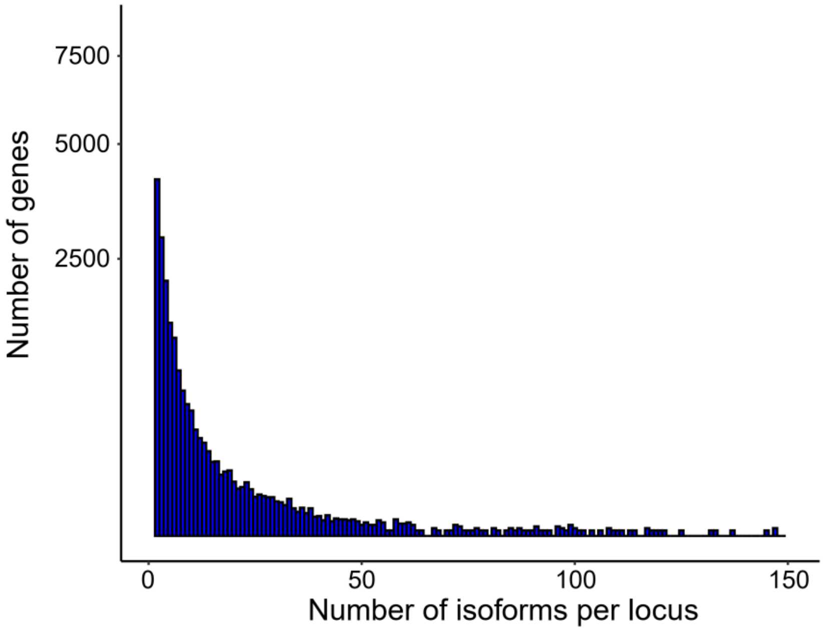

Figure S4. The distribution of isoform numbers for each gene.

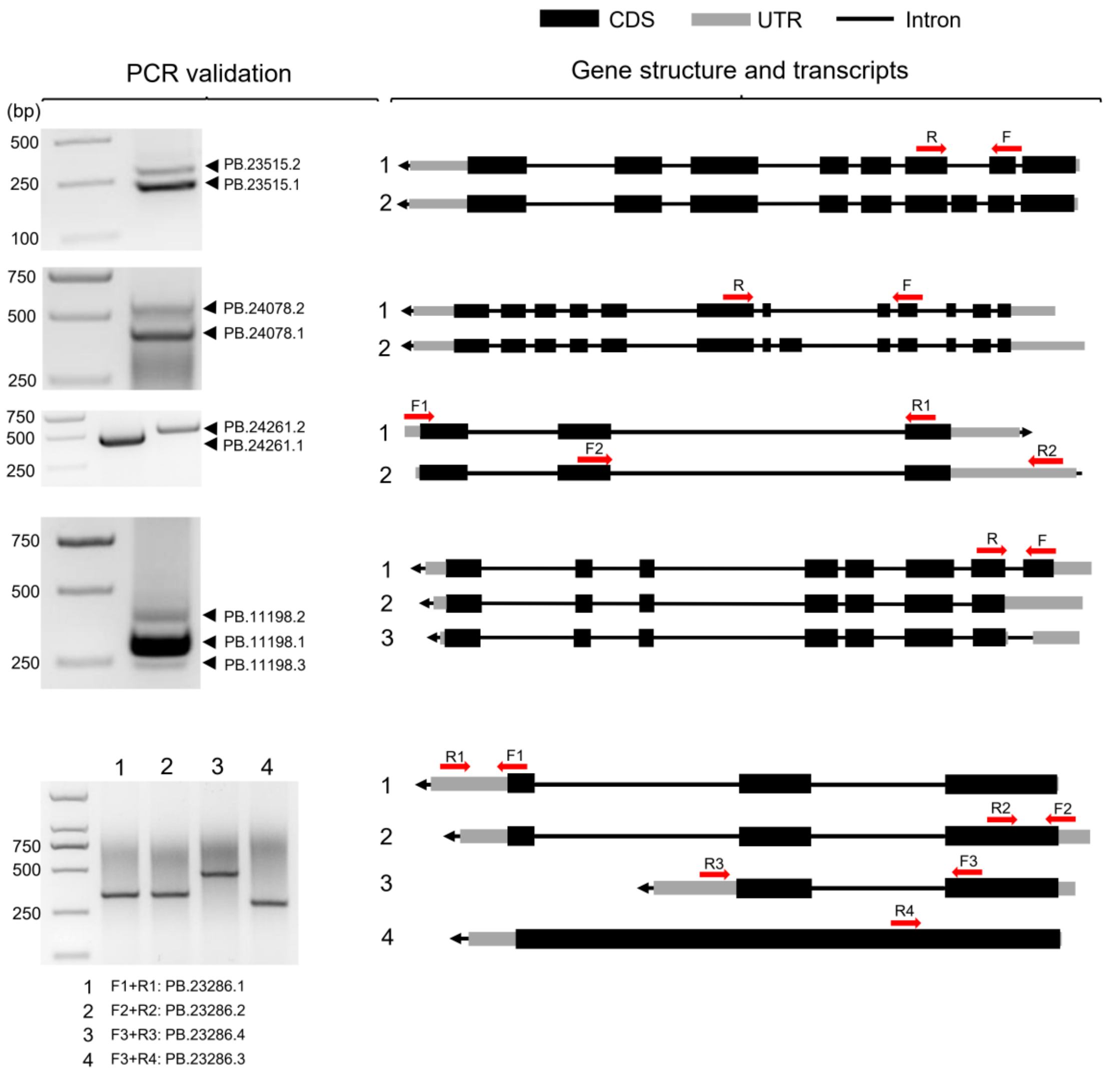

Figure S5. RT-PCR validation of transcripts from Iso-Seq.

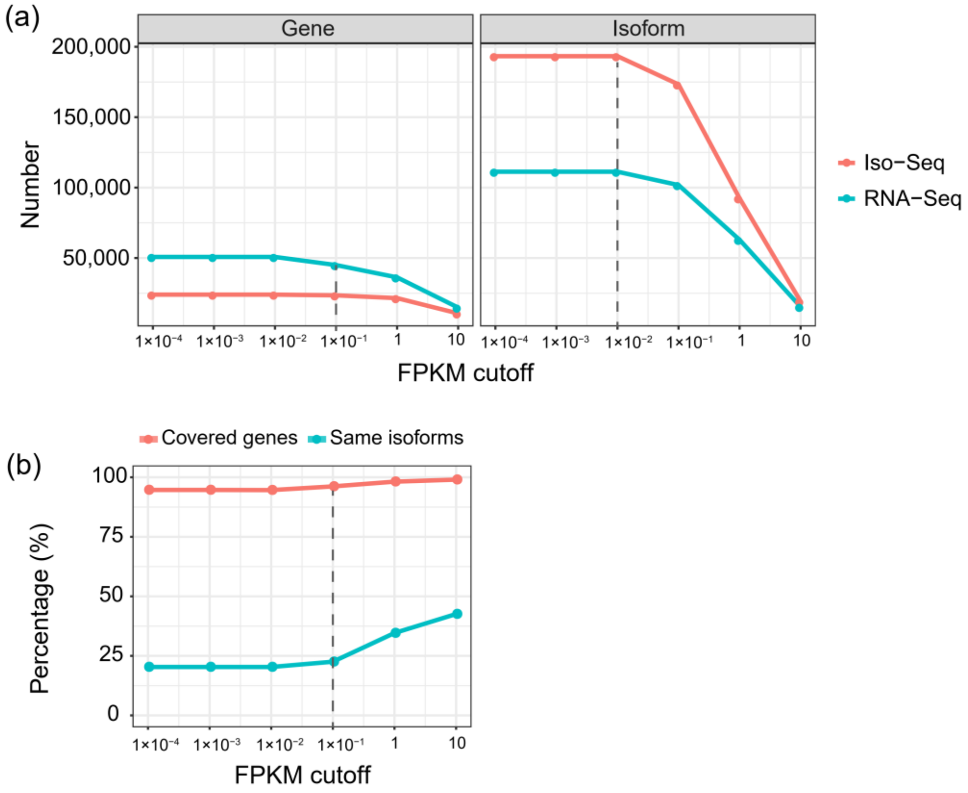

Figure S6. Quantification of Iso-Seq data with RNA-Seq reads, compared to that of reference-based assembled transcripts by RNA-Seq short reads. **(a)** Number of expressed genes and isoforms using different FPKM cutoff values based on Iso-Seq and RNA-Seq assembled transcripts; **(b)** Percentage of identical genes and isoforms by comparing Iso-Seq transcripts with RNA-seq assembled transcripts at different FPKM cutoff values.

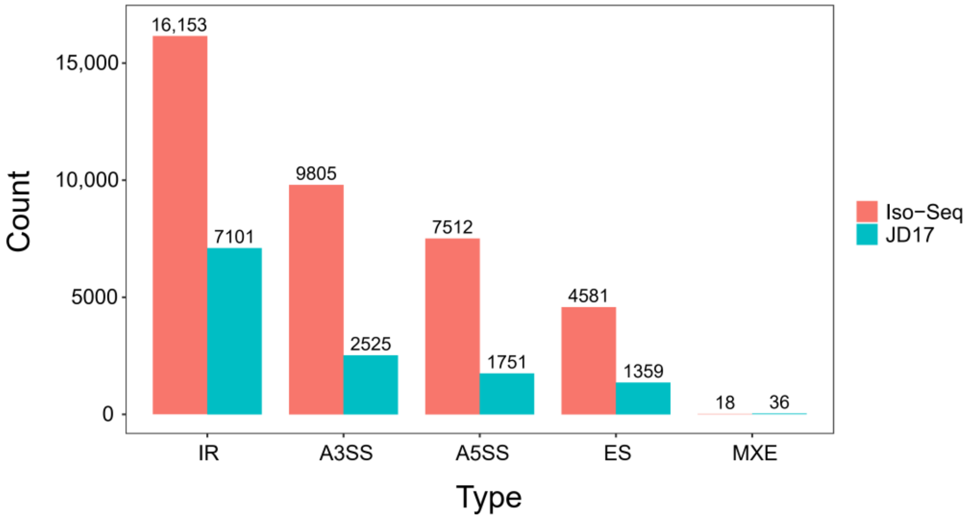

Figure S7. Detection of genome-wide AS events in Iso-Seq and JD17 reference annotation. Five typical AS events were showed. IR: intron retention, A3SS: alternative 3’ splicing site, A5SS: alternative 5’ splicing site, ES: exon skipping, MXE: mutually exclusive exons.

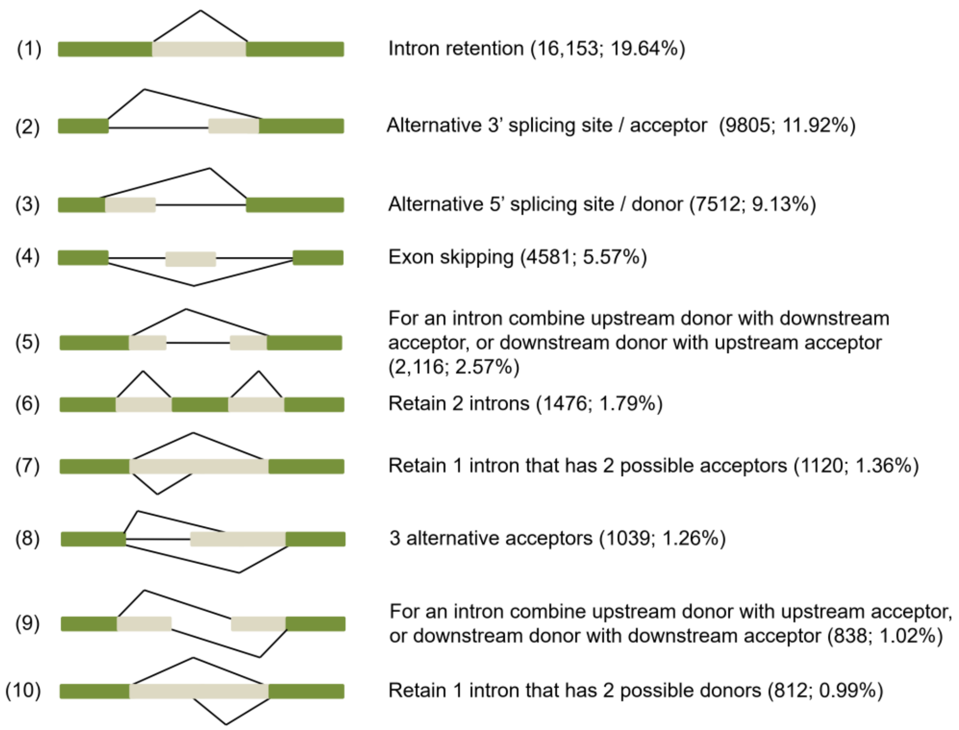

Figure S8. Top 10 most frequent AS events in Iso-Seq data.

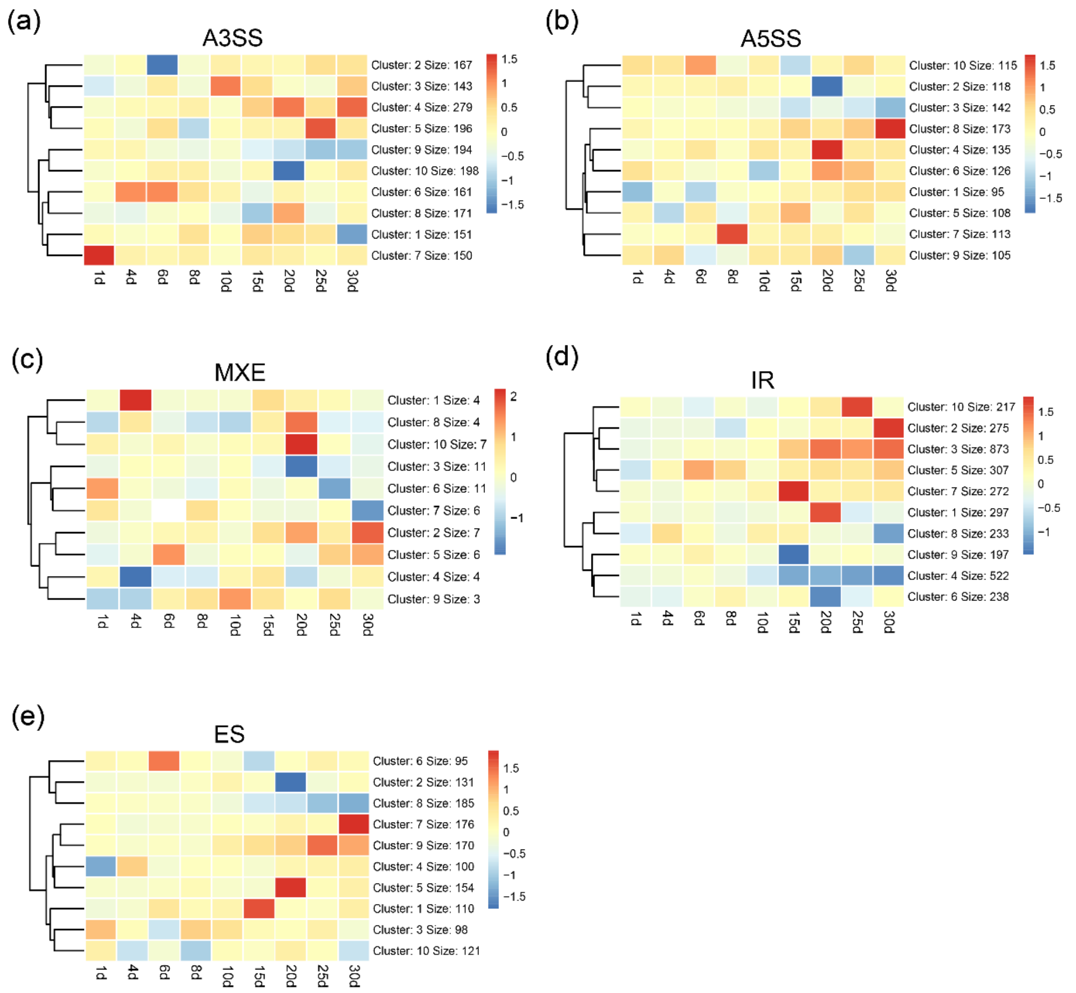

Figure S9. Different AS types showed specific patterns.

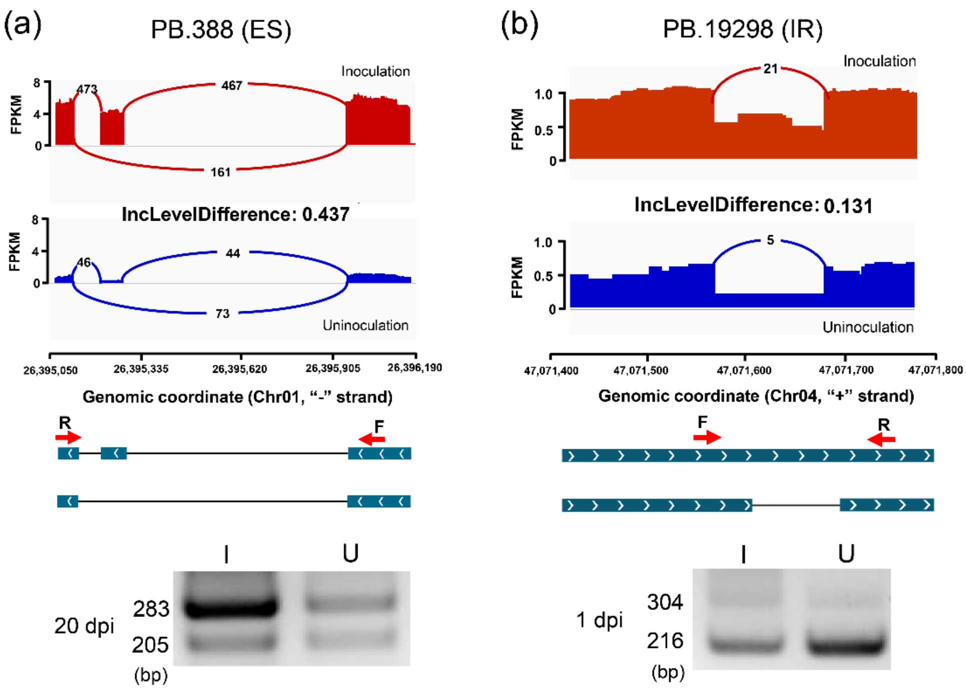

Figure S10. RT-PCR validation of differentially spliced AS events. Validation of ES event (**a**) and IR event (**b**). IncLevelDifference showed the different inclusion level of skipped exon or retained intron between inoculated and uninoculated samples. I and U represent inoculated and uninoculated samples, respectively.

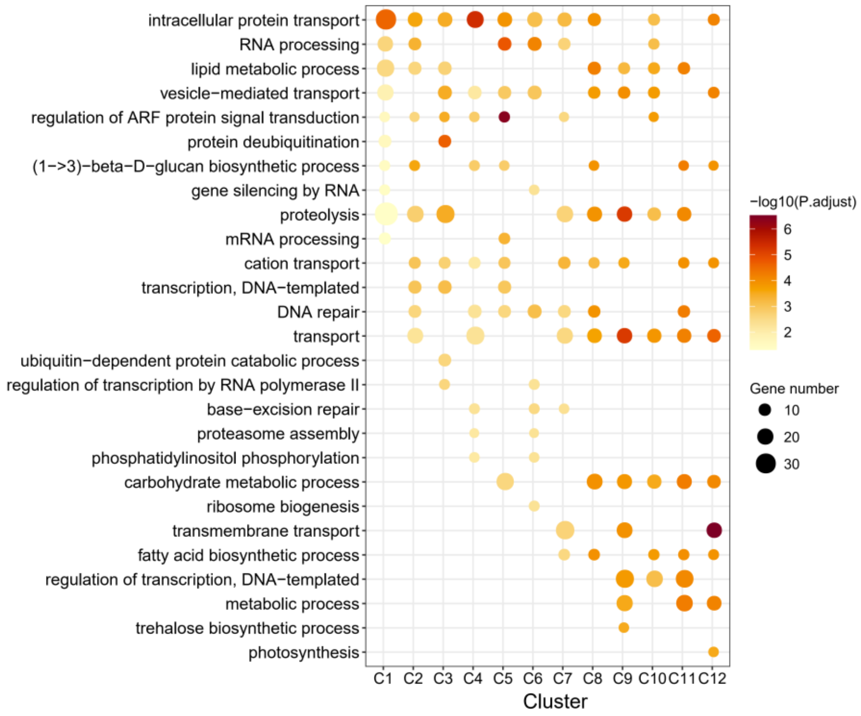

Figure S11. GO enrichment analysis for 12 clusters of differentially expressed transcripts from not differentially expressed genes in Figure 3b. C means Cluster.

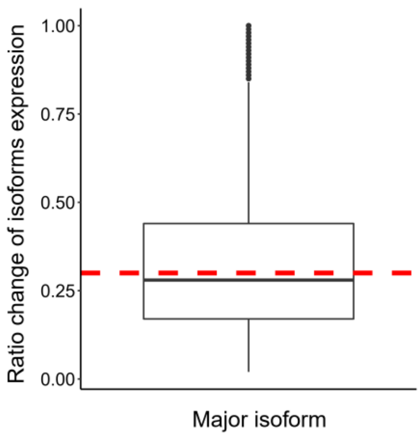

Figure S12. The distribution of isoforms' relative expression ratio changes.

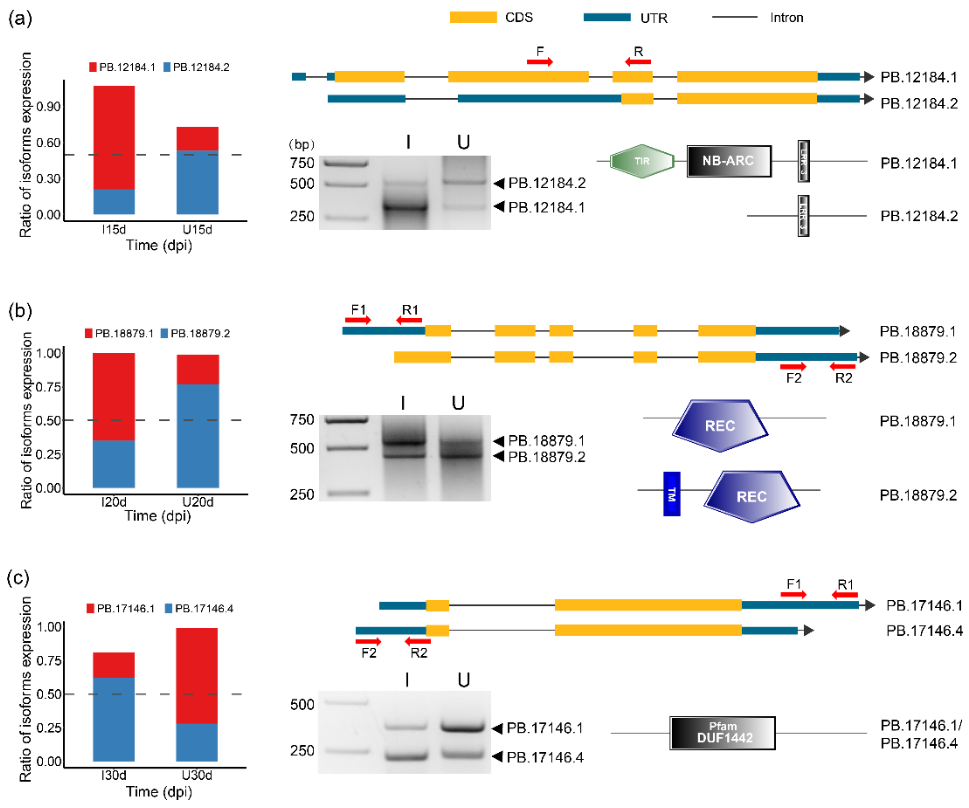

Figure S13. Examples of genes that underwent major isoform switches. Three aspects were compared between two shifted isoforms for each case. Ratio of isoforms expression under inoculated and uninoculated condition at the switched time point, transcripts structures of two variants, and their respective protein domains. RT-PCR confirmed our results of obvious major isoform changes at corresponding time point, and mixed primers were used in (**b**) and (**c**). Pfam DUF1442 represents ankyrin repeat domain, and TM means transmembrane regions. I and U represent inoculated and uninoculated samples, respectively.

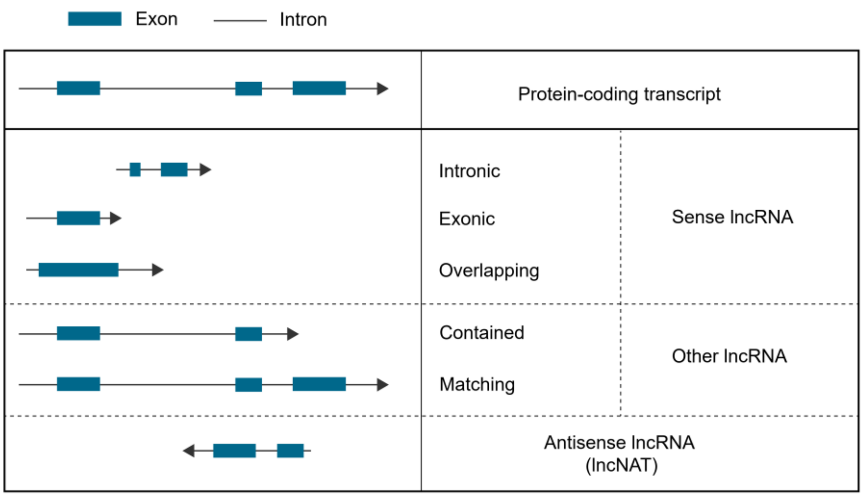

Figure S14. Schematic diagram for classification of lncRNAs.

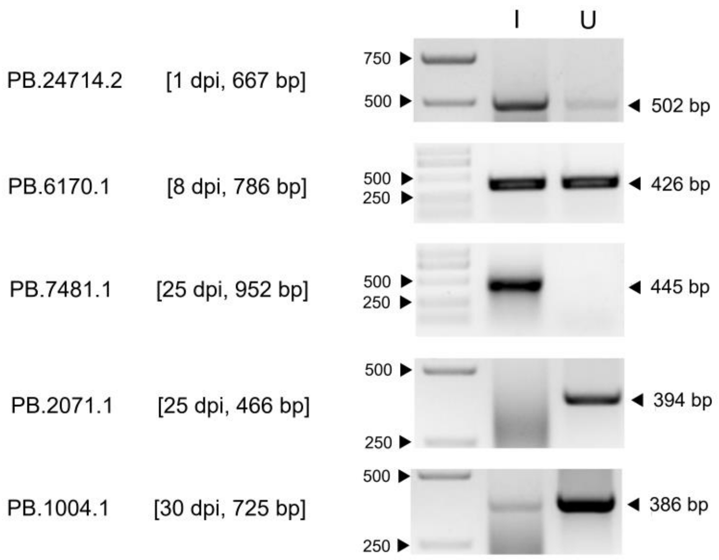

Figure S15. RT-PCR validation of lncRNAs. The values in brackets showed time-points for verification and length of lncRNAs, I: inoculation, U: uninoculation.

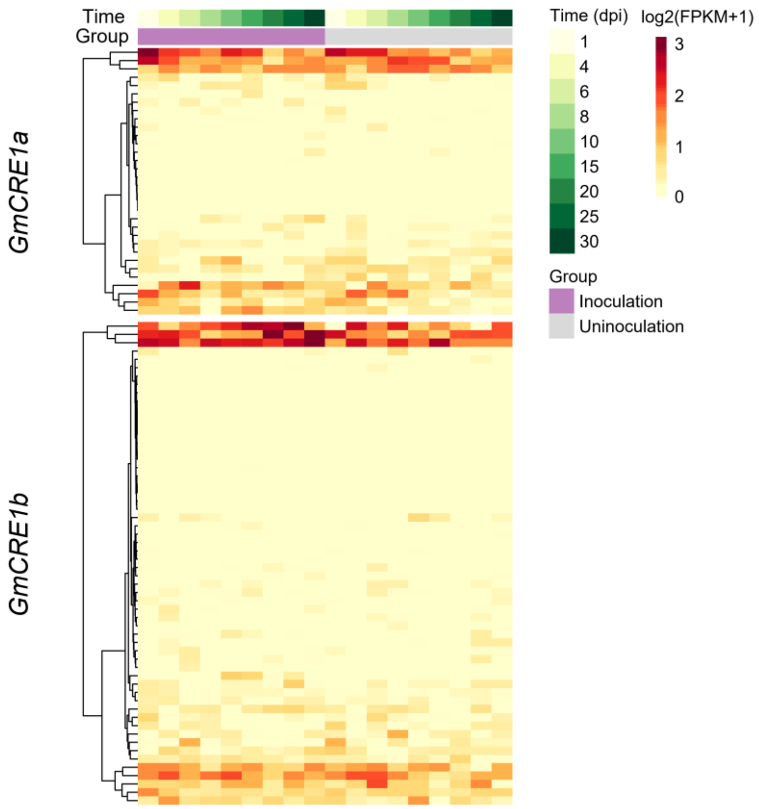

Figure S16. Expression pattern of transcript variants from two soybean cytokinin receptor genes (*GmCRE1a/b*).

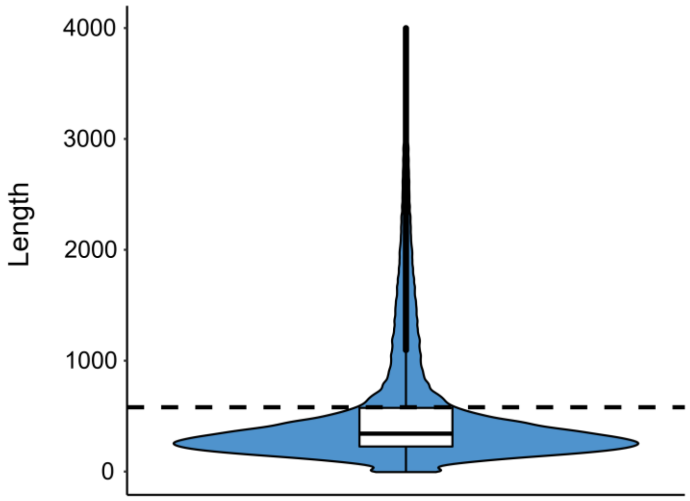

Figure S17. The length distribution of 3’ UTRs in Iso-Seq transcripts. Dash line marks the third quartile of all 3’UTR length (580 nt).
